# Identification of diagnostic hub genes related to neutrophils and infiltrating immune cell alterations in idiopathic pulmonary fibrosis

**DOI:** 10.3389/fimmu.2023.1078055

**Published:** 2023-06-02

**Authors:** Yingying Lin, Xiaofan Lai, Shaojie Huang, Lvya Pu, Qihao Zeng, Zhongxing Wang, Wenqi Huang

**Affiliations:** ^1^ Department of Anesthesiology, The First Affiliated Hospital, Sun Yat-sen University, Guangzhou, China; ^2^ Zhongshan School of Medicine, Sun Yat-sen University, Guangzhou, China

**Keywords:** hub genes, neutrophils, infiltrating immune cell, idiopathic pulmonary fibrosis, immune microenvironment, machine learning, diagnostic model

## Abstract

**Background:**

There is still a lack of specific indicators to diagnose idiopathic pulmonary fibrosis (IPF). And the role of immune responses in IPF is elusive. In this study, we aimed to identify hub genes for diagnosing IPF and to explore the immune microenvironment in IPF.

**Methods:**

We identified differentially expressed genes (DEGs) between IPF and control lung samples using the GEO database. Combining LASSO regression and SVM-RFE machine learning algorithms, we identified hub genes. Their differential expression were further validated in bleomycin-induced pulmonary fibrosis model mice and a meta-GEO cohort consisting of five merged GEO datasets. Then, we used the hub genes to construct a diagnostic model. All GEO datasets met the inclusion criteria, and verification methods, including ROC curve analysis, calibration curve (CC) analysis, decision curve analysis (DCA) and clinical impact curve (CIC) analysis, were performed to validate the reliability of the model. Through the Cell Type Identification by Estimating Relative Subsets of RNA Transcripts algorithm (CIBERSORT), we analyzed the correlations between infiltrating immune cells and hub genes and the changes in diverse infiltrating immune cells in IPF.

**Results:**

A total of 412 DEGs were identified between IPF and healthy control samples, of which 283 were upregulated and 129 were downregulated. Through machine learning, three hub genes (*ASPN, SFRP2, SLCO4A1*) were screened. We confirmed their differential expression using pulmonary fibrosis model mice evaluated by qPCR, western blotting and immunofluorescence staining and analysis of the meta-GEO cohort. There was a strong correlation between the expression of the three hub genes and neutrophils. Then, we constructed a diagnostic model for diagnosing IPF. The areas under the curve were 1.000 and 0.962 for the training and validation cohorts, respectively. The analysis of other external validation cohorts, as well as the CC analysis, DCA, and CIC analysis, also demonstrated strong agreement. There was also a significant correlation between IPF and infiltrating immune cells. The frequencies of most infiltrating immune cells involved in activating adaptive immune responses were increased in IPF, and a majority of innate immune cells showed reduced frequencies.

**Conclusion:**

Our study demonstrated that three hub genes (*ASPN, SFRP2*, *SLCO4A1*) were associated with neutrophils, and the model constructed with these genes showed good diagnostic value in IPF. There was a significant correlation between IPF and infiltrating immune cells, indicating the potential role of immune regulation in the pathological process of IPF.

## Introduction

Idiopathic pulmonary fibrosis (IPF) is a kind of chronic, progressive and irreversible fibrotic interstitial lung disease (ILD) of unknown etiology (1). If IPF is left untreated, continuous disease progression can ultimately lead to destruction of the lung tissue structure, decreased lung compliance and even respiratory failure and death. It was reported that the average life expectancy of IPF patients is only 3-5 years after diagnosis without prompt treatment (2). Thus, early diagnosis and timely treatment are very important. IPF is diagnosed by identifying the pathological pattern of common interstitial pneumonia based on radiological or histological criteria without other evidence of etiology (3). However, it is not easy to exclude other idiopathic interstitial pneumonias and known causes of interstitial lung disease, such as viral infections, chemoradiotherapy, environmental toxicants and chronic inflammatory diseases (4). Thus, it is meaningful to search for specific indicators or construct a diagnostic model for identifying IPF.

Although IPF is characterized by sustained epithelial cell damage, fibroblast activation and excessive deposition of the extracellular matrix in the lung parenchyma, the pathological mechanism of fibrosis in IPF is still poorly understood (5). Substantial evidence from preclinical and clinical studies suggests that immune dysfunction contributes to the progression of IPF (6, 7). However, the roles of different immune dysfunctions and diverse immune cells in IPF are currently unclear. In fact, there is no consensus thus far on whether immune regulation is beneficial or harmful in IPF. IPF was originally thought to be a kind of inflammatory disease (8). However, subsequent clinical trials showed that immunosuppressive agents did not stop disease progression and conversely harmed patients with IPF (9). Similarly, it was reported that the decreased activity of several immune pathways in IPF were associated with poor progression-free survival (10). Together, these results demonstrated that the immunosuppressive microenvironment in IPF might accelerate the progression of this disease. However, it was also reported in other studies that an increased proportion or activation of some immune cells influences disease progression and accelerates the deterioration of lung function in patients with IPF (11–13).

In this study, we searched for hub genes for IPF diagnosis, which might show promise as specific indicators of IPF. Animal models and a meta-GEO cohort were established to further verify the differential expression of these genes. Then, we constructed a diagnostic model of IPF using the hub genes through machine learning and validated the reliability of the model in all the GEO cohorts, which all met the inclusion criteria. Furthermore, the analysis of diverse infiltrating immune cells was performed to explore the immune microenvironment in IPF. By establishing links between hub genes and infiltrating immune cells, we hoped to explore which immune cells might play an important role in IPF.

## Methods

### Data acquisition and processing

**Figure 1:**
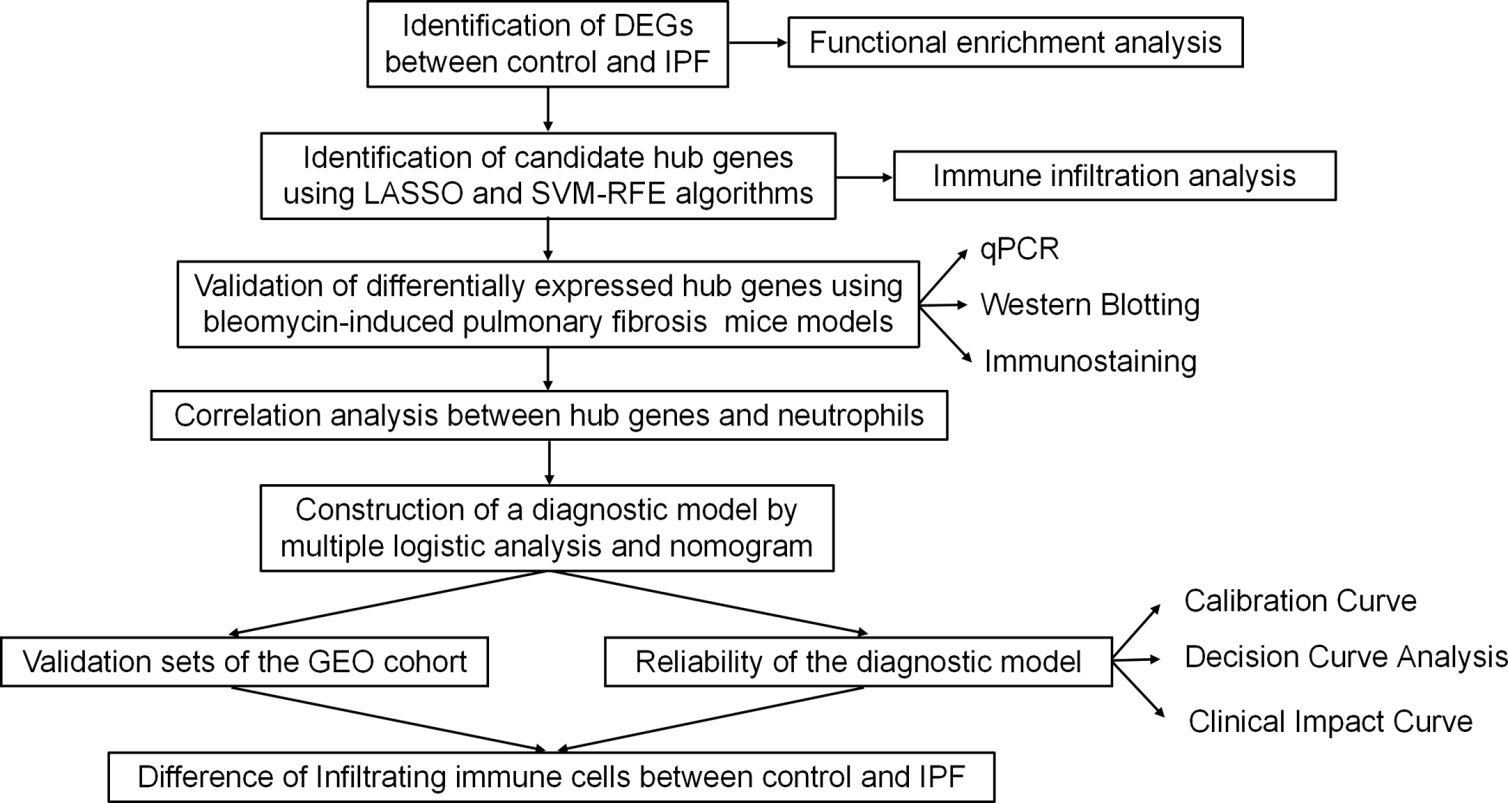
Study flowchart. DEGs, differentially expressed genes; qPCR, quantitative polymerase chain reaction; GEO, Gene Expression Omnibus.

Our study flow chart is shown in **Figure 1**. All expression profiling data generated with arrays used in this study were downloaded from the GEO database (https://www.ncbi.nlm.nih.gov/geo/). Our inclusion criteria were as follows: more than 30 lung samples from *Homo sapiens* and concurrent inclusion of both IPF patients and healthy controls. According to the inclusion criteria, five GEO datasets including GSE32537 (healthy controls=50, IPF=119), GSE17978 (healthy controls=20, IPF=38), GSE53845 (healthy controls=8, IPF=40), GSE110147 (healthy controls=11, IPF=27), and GSE10667 (healthy controls=15, IPF=31) were identified. After background calibration and normalization of the data, the differentially expressed genes (DEGs) were screened by the Limma package according to the following criteria: |log2-fold change| > 1 and adjusted *P* value <0.05.

### Gene enrichment analysis

Gene Ontology (GO) is an international standardized gene function classification system to comprehensively describe the properties of genes and gene products in an organism, including biological process (BP), molecular function (MF) and cellular component (CC) terms (14). The Kyoto Encyclopedia of Genes and Genomes (KEGG) is a well-known database for pathway-associated investigation of genes (15). To explore functions in IPF, we performed GO and KEGG enrichment analyses of the DEGs identified between IPF and healthy control samples. Disease Ontology (DO) is a database established in 2003 that includes common and rare diseases (16). In this study, we also performed DO enrichment analysis to explore the diseases favored by the DEGs.

### Construction and validation of the diagnostic model

Among the included datasets, GSE32537 had the largest number of samples, so we selected it as our training set. First, LASSO regression and SVM-RFE machine learning algorithms were performed to identify candidate hub genes for a diagnostic model (17). Then, we constructed the diagnostic model using hub genes and calculated the risk score of the model using multiple logistic regression analysis. The model was presented as a nomogram. ROC curve analysis was subsequently performed to evaluate the diagnostic value of the nomogram. To further validate the reliability of the diagnostic model, we integrated GSE17978 and GSE53845 into one dataset, creating one dataset with more than 100 samples, as our main validation cohort. To eliminate batch effects in the merged dataset, we applied the Bioconductor “SVA” R package. We also used the GSE110147 and GSE10667 datasets as external validation cohorts. Calibration curve (CC) analysis, decision curve analysis (DCA) and clinical impact curve (CIC) analysis were also performed to evaluate the clinical diagnostic value of the model.

### Infiltrating immune cell analysis

In this study, we used the Cell Type Identification by Estimating Relative Subsets of RNA Transcripts (CIBERSORT) algorithm to identify the proportions of 22 immune cell types in lung samples from IPF patients and healthy controls (18). Correlation analyses between infiltrating immune cells and hub genes were also performed to explore which kinds of infiltrating immune cells were the main participants in the development of IPF. Differences were considered significant when the CIBERSORT output *P* value < 0.05. We also performed correlation analysis of the different types of infiltrating immune cells in IPF (19).

### Animal experiments

Our animal experiments were approved by the Ethics Committee of Sun Yat-sen University. All C57BL/6 mice were fed in a colony room in the Sun Yat-sen University Animal Center with a 12:12 hour light/dark cycle. We injected mice with bleomycin (Teva Pharmaceutical; 3 U/kg) or an equal volume of PBS intratracheally when they were eight weeks old. On day 21 after injection, all mice were harvested, and lung samples were collected for further analysis.

### Histopathology, immunofluorescence and immunohistochemistry staining analysis

Mouse lung samples were embedded in paraffin and cut into sections. Then, we performed hematoxylin and eosin (H&E), Masson trichrome and Sirius Red staining to analyze pulmonary fibrosis. As previously described (20), the method for immunofluorescence (IF) staining of mouse lung samples was as follows: lung samples were incubated with appropriate primary antibodies (**Supplementary Table S1**) overnight at 4°C after dewaxing and antigen retrieval. Then, we stained the sections with a secondary antibody (**Supplementary Table S1**) that recognized the primary antibody for 40 min at room temperature. Stained sections were imaged with a Zeiss 800 laser scanning confocal microscope. The immunohistochemistry (IHC) method involves several steps. First, antigen retrieval and blocking were performed to enhance the visibility of target antigens. Next, primary antibodies, followed by secondary antibodies (**Supplementary Table S1**), were sequentially incubated with lung tissues. Subsequently, diaminobenzidine (DAB) was applied as a chromogen to stain the target antigens. The presence of a brown signal is considered indicative of positive staining. As suggested in other study (21), the analysis of occupied area was conducted by selecting brown regions using a consistent threshold value within the macro function of ImageJ software (NIH). The findings were expressed as the percentage of the area occupied by positive staining relative to the total area in each specimen.

### RNA extraction and quantitative real-time PCR

RNA was extracted from lung samples using TRIzol reagent (Molecular Research Center, Inc.) and reverse transcribed into cDNA with the RevertAid First Strand cDNA Synthesis Kit (Thermo Fisher Scientific, K1622). Then, we performed quantitative real-time PCR (qPCR) using LightCycler480 SYBR Green I Master Mix (Roche, 4887352001-1). 18S served as the internal control. Differences were considered statistically significant with a *P* value < 0.05 using an independent-sample t test or the Mann-Whitney U test. All primer sequences used in this study are listed below: mouse *Aspn* forward 5’-TCCTCTGACAAGGTTGGACT-3’, and reverse 5’-AGAGAGTTGTCGTCATCATCGT-3’; mouse *Sfrp2* forward 5’-CGTGGGCTCTTCCTCTTCG-3’, and reverse 5’-ATGTTCTGGTACTCGATGCCG-3’; mouse *Slco4a1* forward 5’-CGATCTGCACAGCTACCAGAG-3’, and reverse 5’-GCTGACGAAGGTAAGGCATAG-3’; And mouse *18s* forward 5’-GTGACGTTGACATCCGTAAAGA-3’, and reverse 5’-GCCGGACTCATCGTACTCC-3’.

### Western blotting

Mouse lung samples were prepared using RIPA buffer (Beyotime, P0013B). The total protein concentration was assessed using the Pierce BCA Protein Assay (Thermo Fisher, 23227). After electrophoresis, proteins were transferred to PVDF membranes (Millipore). Then, the target proteins were immunoblotted with specific antibodies. The signal intensity of protein bands was visualized with a chemiluminescent substrate (Millipore). All protein bands from three independent blots were quantified using ImageJ software. The corresponding primary and secondary antibodies utilized are listed below: rabbit anti-Asporin (Invitrogen, PA5-28124), rabbit anti-SFRP2 (Affifinity Biosciences, DF4451), rabbit anti-SLCO4A1 (XY-Bioscience, XY12713), rabbit anti-GAPDH (Cell Signaling Technology, D16H11), and anti-rabbit IgG HRP-linked Ab (Cell Signaling Technology, 7074).

### Statistical analysis

All statistical analyses performed in this study were conducted with R software, version 4.1.3 (http://www.r-project.org). An independent-sample t test was applied to validate the differences between two groups if the continuous variables were normally distributed; otherwise, we used the Mann-Whitney U test. A *P* value < 0.05 was considered statistically significant.

## Results

### Identification of differentially expressed genes between IPF and healthy control samples and functional enrichment analysis of IPF

**Figure 2:**
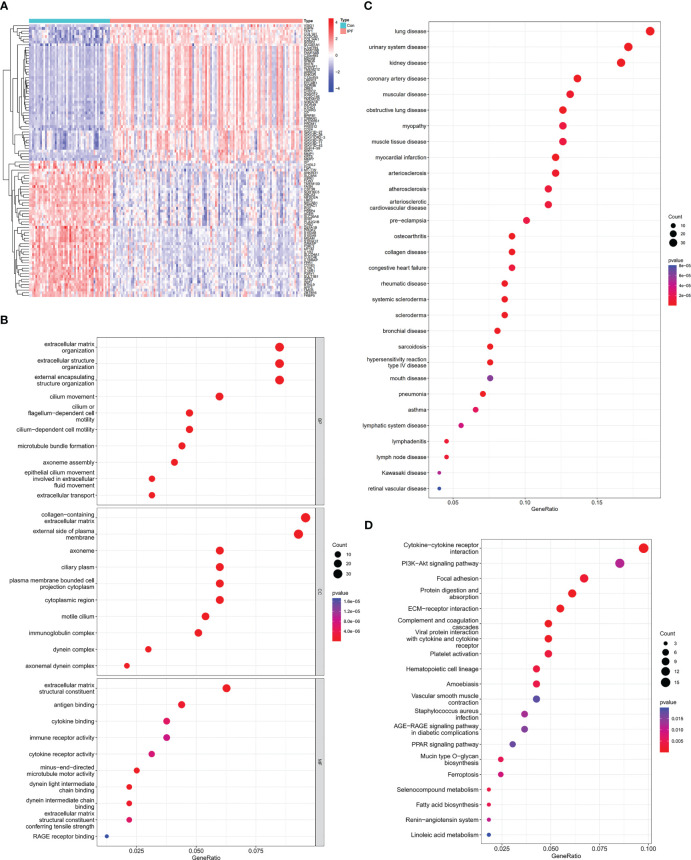
Identification of DEGs between IPF and healthy control samples and functional enrichment analysis of IPF. **(A)** Heatmap of DEGs identified between IPF and healthy control samples. Red represents upregulated genes, and blue represents the opposite. **(B)** GO enrichment analysis of DEGs. **(C)** DO enrichment analysis of DEGs. **(D)** KEGG enrichment analysis of DEGs.

First, we downloaded RNA sequences of lung samples from the GEO database (GSE32537, including 119 IPF subjects and 50 healthy controls). After bioinformatic analysis with Limma, a total of 412 DEGs were identified between the IPF and healthy control samples, of which 283 were upregulated and 129 were downregulated (**Figure 2A**; **Supplementary Figure S1A**).

Then, we investigated the potential biological functions of the DEGs through GO enrichment analysis. We found that the DEGs were mainly enriched in the extracellular matrix (ECM), extracellular structure and external encapsulating structure organization for BP terms. With regard to CC terms, these genes were mainly involved in collagen-containing ECM and external side of plasma membrane. In the MF category, they were strongly related to ECM structural constituent (**Figure 2B**). The GO enrichment analysis revealed that IPF was tightly linked to the pathological ECM deposition process. Moreover, DO enrichment analysis showed that these DEGs were mainly enriched in lung diseases. Together, these results indicated that these DEGs were reliable for subsequent IPF research (**Figure 2C**). Through KEGG enrichment analysis, we also found that IPF was strongly related to cytokine-cytokine receptor interaction and focal adhesion signaling pathways (**Figure 2D**).

### Identification of candidate hub genes through machine learning

**Figure 3:**
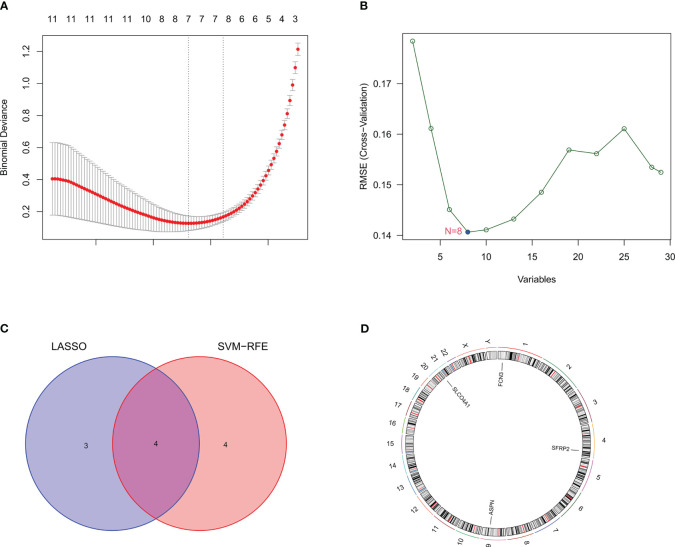
Identification of candidate hub genes through machine learning. **(A)** The candidate hub genes screened by LASSO regression. The nadir of the curve corresponds to the suitable genes of the diagnostic model. **(B)** The candidate hub genes screened by the SVM-RFE machine learning algorithm. The nadir of the curve corresponds to the suitable genes of the diagnostic model. **(C)** The Venn diagram shows the intersection of the genes obtained by LASSO regression and SVM-RFE machine learning algorithm. **(D)** The four candidate genes and their chromosomal locations.

We identified the candidate hub genes of a diagnostic model by LASSO regression combined with SVM-RFE machine learning algorithms. LASSO regression identified seven potential genes (*ASPN, SFRP2, SLCO4A1, IL1R2, MMP7, FCN3*, and *CP*) (**Figure 3A**), and the SVM-RFE algorithms screened eight potential genes (*ASPN, SFRP2, FCN3, COL14A1, MGAM, SLCO4A1, CD24*, and *SPATA18*) (**Figure 3B**). By taking the intersecting genes, we identified four candidate hub genes (*ASPN, SFRP2, FCN3*, and *SLCO4A1*) (**Figures 3C, D**). Each gene showed reliable classification between IPF and healthy control samples by ROC curve analysis (**Supplementary Figures S1B–E**).

### Candidate hub genes showed significant expression differences between fibrotic lungs and control lungs in bleomycin-induced pulmonary fibrosis model mice

**Figure 4:**
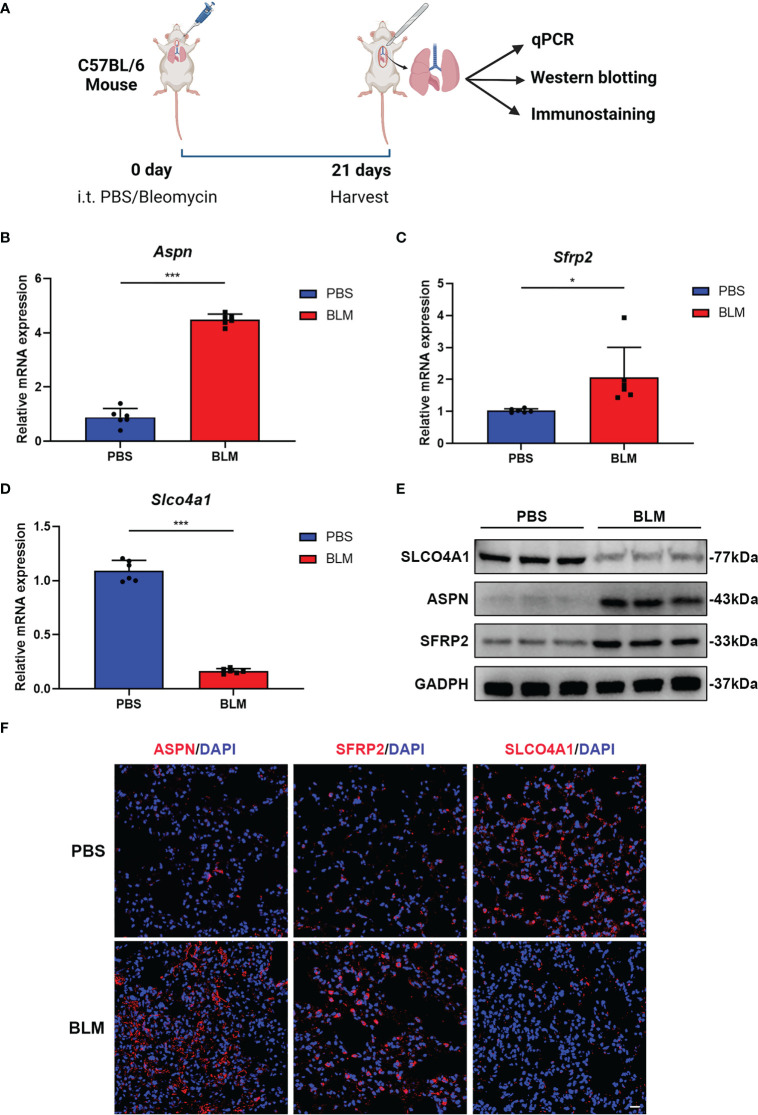
*ASPN*, *SFRP2* and *SLCO4A1* showed significant expression differences between fibrotic lungs and control lungs in bleomycin-induced pulmonary fibrosis model mice. **(A)** Diagram of animal model construction and evaluation. **(B-D)** qPCR analysis of the relative mRNA (*Aspn*, *Sfrp2* and *Slco4a1*) expression levels in bleomycin-induced pulmonary fibrosis model mice (n = 6 mice per group). **(E)** Western blot analysis of the relative protein (*Aspn*, *Sfrp2* and *Slco4a1*) expression levels in bleomycin-induced pulmonary fibrosis model mice (n = 3 mice per group). **(F)** Representative immunofluorescence images showing the locations of *ASPN*, *SFRP2* and *SLCO4A1* in the lungs of bleomycin-treated or PBS-treated mice. Scale bars, 50 µm. All the experiments have been repeated three times and the data are presented as the mean ± SD. *P < 0.05, **P < 0.01, and ***P < 0.001.

To further verify the differential expression of candidate hub genes between IPF and control samples, we then established a bleomycin-induced pulmonary fibrosis mouse model and detected the expression of *SLCO4A1, ASPN* and *SFRP2* between fibrotic lung samples and control samples from model mice (**Figure 4A**). Since *FCN3* is a pseudogene in mice, we were unable to validate it in the mouse model (22). As shown by H&E, Masson trichrome, and Sirius Red staining, there was significant collagen deposition and fibrosis in the lung samples from bleomycin-induced model mice compared with those from PBS-treated mice (**Supplementary Figure S2A**). By qPCR and western blotting, we found that the mRNA and protein levels of *ASPN* and *SFRP2* were upregulated in lung samples from bleomycin-induced model mice, while *SLCO4A1* was downregulated (**Figures 4B–E**). Similarly, IF staining also produced the same findings (**Figure 4F**). Overall, our hub genes showed significant differential expression between fibrotic lungs and control lungs, and their change trends were consistent with those in the meta-GEO cohort including GSE32537, GSE17978, GSE53845, GSE110147 and GSE10667 (**Supplementary Figures S2B–D**).

### Candidate hub genes showed strong correlations with a decline in the neutrophil level

**Figure 5:**
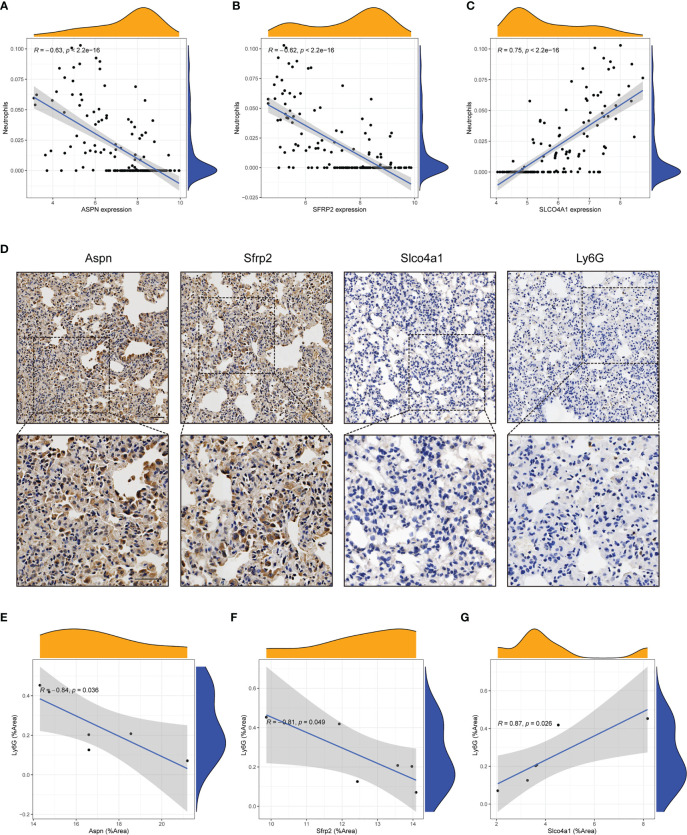
Candidate hub genes showed strong correlations with neutrophils. **(A-C)** Correlation analysis between neutrophils and candidate hub genes (*ASPN*, *SFRP2* and *SLCO4A1*) in the GEO cohort. **(D)** Representative immunohistochemical images of *Aspn*, *Sfrp2*, *Slco4a1* and *Ly6G* in the lung tissues of bleomycin-treated mice (n = 6). Scale bars, 50µm. **(E-G)** Correlation analysis between the expression of candidate hub genes (*Aspn*, *Sfrp2* and *Slco4a1*) and *Ly6G* in the lung tissue of bleomycin-treated mice (n = 6). All the experiments have been repeated three times and the data are presented as the mean ± SD.

To explore the relationships between candidate hub genes and infiltrating immune cells, the CIBERSORT algorithm was used. We found that all four candidate hub genes showed significant correlations with immune cells (**Supplementary Figure S3A–D**), including neutrophils, regulatory T cells, and resting mast cells. Among the different types of infiltrating immune cells, the expression of all four hub genes had the strongest link with a decline in the neutrophil level. The expression of *ASPN (P*<0.001, R=-0.63) and *SFRP2* (*P*<0.001, R=-0.62) had a negative relationship with neutrophils (**Figures 5A, B**), while *SLCO4A1* (*P*<0.001, R=0.75) and *FCN3* (*P*<0.001, R=0.58) had a positive relationship with neutrophils (**Figure 5C**; **Supplementary Figure S3E**). *Ly6G* has been widely acknowledged as a key marker for identifying neutrophils in murine models (23). In order to further substantiate the relationship between hub genes and neutrophils, we conducted an analysis of the correlation between the expression of hub genes and *Ly6G* using IHC analysis in a bleomycin-induced mouse model (**Figure 5D**). Our findings revealed a significant negative relationship between the expression of *Aspn* and *Sfrp2* and the expression of *Ly6G* (**Figures 5E, F**), consistent with the aforementioned results. Conversely, the expression of *Slco4a1* demonstrated a positive relationship (**Figure 5G**). Collectively, these results reinforce our previous findings and suggest a potential decrease in neutrophil levels within the immune microenvironment of IPF.

### Construction of a diagnostic model using hub genes

**Figure 6:**
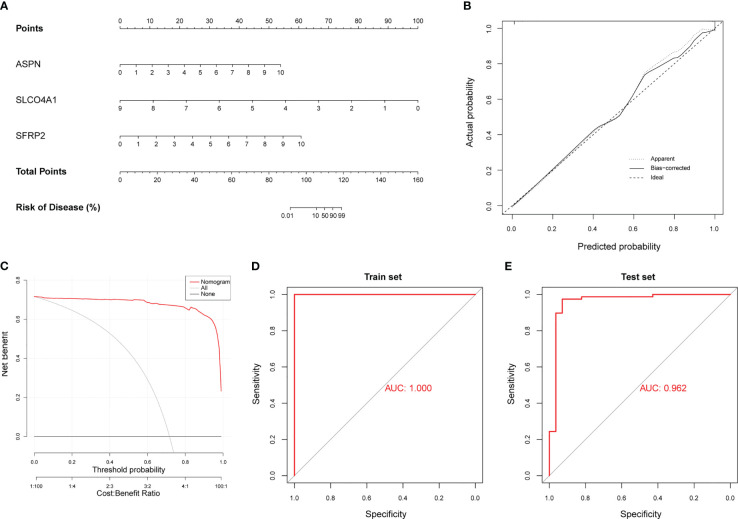
Construction of a diagnostic model using neutrophil-associated hub genes. **(A)** The nomogram presents the diagnostic model constructed with the three hub genes. **(B)** Calibration curve (CC) of the diagnostic model. **(C)** Decision curve analysis (DCA) of the diagnostic model. **(D)** ROC curve for the training cohort (GSE32537). **(E)** ROC curve for the validation cohort (merged dataset containing GSE17978 and GSE53845).

Considering that we could not validate the differential expression of FCN3 in animal models, we ultimately identified three hub genes (*ASPN, SFRP2 and SLCO4A1*) to construct a diagnostic model for IPF. The model that incorporated the above three hub genes was developed using multivariate logistic regression analysis and is presented as a nomogram (**Figure 6A**). Furthermore, we obtained a risk score based on the expression levels of the three hub genes, where the risk score = -5.6339 + (the expression level of ASPN × 0.8534) + (the expression level of SFRP2 × 0.7400) + (the expression level of SLCO4A1 × -0.8030). As shown in a CC, the nomogram for the classification of IPF and healthy control samples showed good agreement between prediction and reality (**Figure 6B**). DCA demonstrated that patients could benefit from the diagnostic model developed with the three hub genes at threshold probabilities from 0 to 1 (**Figure 6C**). Similarly, a CIC showed that the predicted number of high-risk patients was very close to the actual number of high-risk patients, further affirming that our model had clinical diagnostic value for IPF (**Supplementary Figure S3F**). Through ROC curve analysis, we found that the AUC of the diagnostic model was 1.000 for the training set (**Figure 6D**). To further validate the reliability of the diagnostic model, we merged two external datasets into a validation cohort (GSE17978 and GSE53835) that included 78 IPF lung samples and 28 healthy control lung samples. The AUC was 0.962 (**Figure 6E**). In regard to the other external cohorts, the AUCs of GSE110147 and GSE10667 were 0.985 and 0.925, respectively (**Supplementary Figure S3G, H**). Overall, it was suggested that our diagnostic model had a strong ability to distinguish IPF patients from healthy controls.

### Significant correlations between IPF and infiltrating immune cells

As shown above, all three hub genes had good diagnostic value for IPF and showed significant correlations with immune cells, especially neutrophils. To further explore the immune microenvironment in IPF, we performed immune cell infiltration analysis between IPF and healthy control samples using the meta-GEO cohort generated by merging five datasets, namely, GSE32537, GSE17978, GSE53845, GSE110147 and GSE10667. The proportions of different immune cells are shown in **Supplementary Figure S4A**, and we found that there was a significant difference in immune cells between IPF and healthy control samples (**Supplementary Figure S4B**), including differences in naïve B cells (*P*<0.001), memory B cells (*P*<0.001), plasma cells (*P*<0.001), naïve CD4 T cells (*P*<0.001), resting memory CD4 T cells (*P*<0.001), activated CD4 memory T cells (*P*<0.001), follicular helper T cells (*P*=0.024), gamma delta T cells (*P*=0.003), resting NK cells (*P*<0.001), monocytes (*P*<0.001), M0 macrophages (*P*=0.039), resting dendritic cells (*P*<0.001), activated dendritic cells (*P*=0.012), resting mast cells (*P*<0.001), eosinophils (*P*<0.001) and neutrophils (*P*<0.001). As shown above, the proportions of most infiltrating immune cells involved in activating an adaptive immune response were increased, and the frequencies of a majority of innate immune cells were reduced in IPF. By analyzing the correlations of different infiltrating immune cells, it was shown that resting memory CD4 T cells had the strongest negative correlation with CD8 T cells (R=-0.57) and neutrophils showed a negative correlation with resting mast cells in IPF lung samples (**Supplementary Figure S4C**). All of the above results showed that the changes in diverse infiltrating immune cells might have roles in the pathogenesis of IPF.

## Discussion

IPF is a progressive and irreversible interstitial lung disease with high mortality and limited treatment options. Although it can be diagnosed by the pathological pattern of usual interstitial pneumonia based on radiological or histological examination, it lacks specific diagnostic indicators that distinguish it from other interstitial lung diseases. Thus, searching for specific indicators or constructing a diagnostic model for identifying IPF is meaningful (24).

In this study, we have identified a total of 412 DEGs between lung samples from patients with IPF and healthy controls. GO enrichment analysis revealed that these DEGs are strongly associated with ECM-related functions, which is in line with the pathological characteristics of IPF. IPF is characterized by the replacement of healthy tissue with excessive ECM (4), leading to elevated biomechanical stiffness and altered cellular behavior, ultimately contributing to aberrant lung remodeling and disease pathogenesis (25–27). Additionally, KEGG pathway analysis revealed that these DEGs primarily participate in cytokine-cytokine receptor interaction and focal adhesion signaling pathways. Both of these pathways are known to play crucial roles in IPF pathogenesis. Integrins, which are the main receptors for cell adhesion to ECM proteins, can promote myofibroblast differentiation and disease pathogenesis (28). Moreover, integrins also activate transforming growth factor-beta (TGF-β), which plays a crucial role in promoting pro-fibrotic cytokine secretion and perpetuating fibrogenesis (29–31).

Furthermore, we found that *ASPN*, *SFRP2* and *SLCO4A1* were differentially expressed between IPF and healthy control samples in the GEO cohorts. Our results were further validated in a mouse model of bleomycin-induced pulmonary fibrosis. *ASPN*, a member of the small leucine-rich proteoglycan family (32), has been shown to encode extracellular matrix proteins (33). In some studies, *ASPN* was also shown to be differentially expressed in lung tissue between IPF patients and healthy controls (34, 35), which was consistent with our findings. Moreover, in our previous study, we demonstrated that *ASPN* could accelerate pulmonary fibrosis by promoting myofibroblast differentiation induced by TGF-β (36) and play an important role during the pathological process of IPF. *SFRP2* was also one of the hub genes in our diagnostic model. *SFRP2* was reported to prompt the development of fibrosis (37). Previous studies have demonstrated that *SFRP2* facilitates the proliferation of cardiac fibroblasts by activating the Wnt/β-catenin pathway and participates in myocardial fibrosis and cardiac remodeling (38, 39). The differentiation of fibroblasts into myofibroblasts is an important pathological characteristic of IPF (2, 5). Progenitors of myofibroblasts have high expression of *SFRP2*, which indicates the important role of *SFRP2* in the differentiation of myofibroblasts (40, 41). Whether *SFRP2* contributes to the development of IPF and the associated mechanism are worthy of further exploration. *SLCO4A1* is a solute carrier organic anion transporter family member. Previous studies have shown that *SLCO4A1* promotes tumorigenesis and the progression of different carcinomas (42, 43), but the relationship between *SLCO4A1* and fibrosis has rarely been reported.

By combining LASSO regression and machine learning, we constructed a diagnostic model using three hub genes. Then, we validated the diagnostic reliability of the model in all GEO datasets meeting the inclusion criteria. The results showed that our diagnostic model showed reliable classification between IPF and healthy control samples. We also found that our diagnostic model had good clinical diagnostic value through DCA and CIC analysis. Further studies are necessary to validate the feasibility of clinical model application for the diagnosis of IPF.

Considering that immune responses might contribute to the progression of IPF (6, 44, 45), we analyzed infiltrating immune cells in IPF and healthy control lung samples. We found that the immune microenvironment was significantly different between IPF and healthy control lungs. The frequencies of most infiltrating immune cells involved in activating adaptive immune responses, such as memory B cells, plasma cells, activated memory CD4 T cells, follicular helper and gamma delta T cells, were increased in IPF. Conversely, a majority of innate immune cells, such as resting NK cells, monocytes, M0 macrophages, eosinophils and neutrophils, showed decreased frequencies.

Although much progress has been made in understanding innate and adaptive immune responses in IPF, their roles are still unclear (5). According to the majority of studies, it has been concluded that an increase in the frequency or activation of certain T cells and B cells is associated with the progression of IPF (12, 13, 45). T helper 2 (Th2) cytokines, including IL-4, IL-5, IL-9 and IL-13, have been confirmed to play a promotive role in fibrosis (46, 47). Cytokines such as TGF-β, IL-1β, CXC, and CC can be secreted by epithelial cells and recruit T cells to induce the migration of adaptive immune cells and prompt the progression of fibrosis. However, previous studies have also reported that cytokine receptor­like factor 1 could enhance the levels of T helper 1 (Th1) and regulatory T cells, playing an antifibrotic role in the lungs (48). Thus, the roles of diverse adaptive immune cells in IPF might be different. In this study, we found that the levels of some of the adaptive immune cells described above were increased in IPF patients compared with healthy controls. Whether activating adaptive immune cells mainly contributes to the progression of IPF and whether suppression of the adaptive immune response is helpful in patients with IPF needs further exploration.

Innate immune cells can recognize and defend against infections caused by pathogens, mounting resistance to reinfection (49). Previous studies have reported that the activity of several immune pathways was reduced in IPF lungs, which was related to a low-diversity microorganisms (10). Several clinical trials have suggested that immunosuppressive agents are harmful to patients with IPF (9). In this study, we demonstrated that the levels of some innate immune cells, such as resting NK cells, monocytes, M0 macrophages, eosinophils and neutrophils, were decreased in IPF lungs compared with healthy control lungs. Thus, an immunosuppressive environment might be related to innate immune responses in IPF, weakening the resistance of IPF patients to infection and promoting the progression of IPF.

In addition, by analyzing the correlations between hub genes and infiltrating immune cells, we found that all hub genes showed a strong correlation with neutrophils and that their expression levels were negatively correlated with the number of neutrophils. Thus, the involvement of neutrophils might play an important role in the pathogenesis of IPF. As the first line of defense against invading pathogens (50), neutrophils perform a series of protective mechanisms to prevent the spread of infection and inflammation (51). It was reported that there are more neutrophils in pulmonary capillaries than in the systemic circulation, which facilitates their rapid response to infection and inflammation in lung tissue (52). Given this perspective, neutrophils contribute to maintaining homeostasis in the lungs and protect IPF patients from damage caused by invading pathogens (7, 53). However, some studies have suggested that neutrophils and their products promote fibrogenesis in IPF. It was reported that an increased frequency of neutrophils in IPF patients increases the degree of pulmonary fibrosis and is correlated with a poor prognosis (54–56). Neutrophil elastase facilitates pulmonary fibrosis through the activation and proliferation of fibroblasts, inducing their differentiation into myofibroblasts (57, 58). Some studies have indicated that neutrophil extracellular traps also contribute to pulmonary fibrosis (59–61). In fact, the role of neutrophils in IPF is still unknown. Whether these cells exert protective or promotive effects on the pathogenesis of IPF is controversial.

Overall, the effects of diverse immune responses and the roles of different immune cells in IPF are elusive. However, it is clear that immune regulation has both advantages and disadvantages in the progression of IPF. Therefore, general anti-immune or anti-inflammatory therapy alone is obviously not advisable. It might be wiser to explore the roles of different immune responses and immune cells in IPF, and then apply targeted immune regulation to restrain the progression of IPF.

There were still potential limitations in our study. First, although we validated that the three hub genes were differentially expressed between IPF and healthy control samples in the GEO cohort and animal model, we could not determine whether these genes can act as specific indicators for IPF diagnosis. The clinical application of the diagnostic model we constructed still needs more clinical evidence for validation. Second, the effects of different immune cells in IPF could not be demonstrated in our study. The roles of adaptive and innate immune responses in the progression of IPF are still elusive. Although we found that the three hub genes showed strong correlations with neutrophils, whether neutrophils truly contribute to IPF could not be confirmed.

## Conclusion

In this study, through combined study of a GEO cohort and an animal model, we identified three hub genes (*ASPN, SFRP2* and *SLCO4A1*) that were differentially expressed between IPF and healthy control samples. Then, we constructed a diagnostic model for IPF using the three hub genes. Through our validation cohorts and statistical methods to verify the reliability of the model, we demonstrated that our model had good diagnostic value. Furthermore, by analyzing the changes in infiltrating immune cells, we explored the possible roles of different immune responses in IPF. We found that there was a significant correlation between IPF and infiltrating immune cells. The levels of most infiltrating immune cells involved in activating an adaptive immune response were increased in IPF, while those of a majority of innate immune cells were reduced. We then analyzed the relationships between the hub genes and infiltrating immune cells. We found that the expression of all hub genes showed strong correlation with the decline in the neutrophil level.

## Data availability statement

The datasets presented in this study can be found in online repositories. The names of the repository/repositories and accession number(s) can be found in the article/**Supplementary Material**.

## Ethics statement

The animal study was reviewed and approved by the Ethics Committee of Sun Yat-sen University.

## Author contributions

YL, XL and SH designed the research, completed most experiments and wrote the manuscript. LP and QZ analyzed the data and provided further input. WH and ZW generated the research idea and revised the manuscript. All authors contributed to the article and approved the submitted version.

## Supplementary material

Supplementary Figure 1
**(A)** Volcano plot of DEGs between IPF and healthy control samples. **(B-E)** ROC curve for each candidate gene (*ASPN*, *SFRP2*, *SLCO4A1* and *FCN3*).

Supplementary Figure 2
**(A)** Representative hematoxylin and eosin (H&E), Masson’s trichrome and Sirius Red staining images obtained for the lungs of bleomycin-treated or PBS-treated mice. **(B–D)** Differential analysis of mRNA (*ASPN*, *SFRP2* and *SLCO4A1*) expression levels using the meta-GEO cohort.

Supplementary Figure 3
**(A-D)** Correlation analysis between infiltrating immune cells and candidate hub genes (*ASPN*, *SFRP2*, *SLCO4A1* and *FCN3*). **(E)** Correlation analysis between neutrophils and *FCN3*. **(F)** Clinical impact curve (CIC) of the diagnostic model. **(G)** ROC curve of an external validation cohort (GSE110147). **(H)** ROC curve of an external validation cohort (GSE10667).

Supplementary Figure 4 Analysis of infiltrating immune cells in IPF using the meta-GEO cohort. **(A)** The proportions of different immune cells in IPF or healthy control lung samples. **(B)** Differential analysis of infiltrating immune cells between IPF and healthy control lung samples. **(C)** Correlation analysis of different infiltrating immune cells in the immune microenvironment of IPF.
