# Supplementary figures and images for: Identification of diagnostic hub genes related to neutrophils and infiltrating immune cell alterations in idiopathic pulmonary fibrosis
Source: Front Immunol. 2023 Jun 2;14:1078055. doi: 10.3389/fimmu.2023.1078055 (PMC10272521; doi:10.3389/fimmu.2023.1078055)

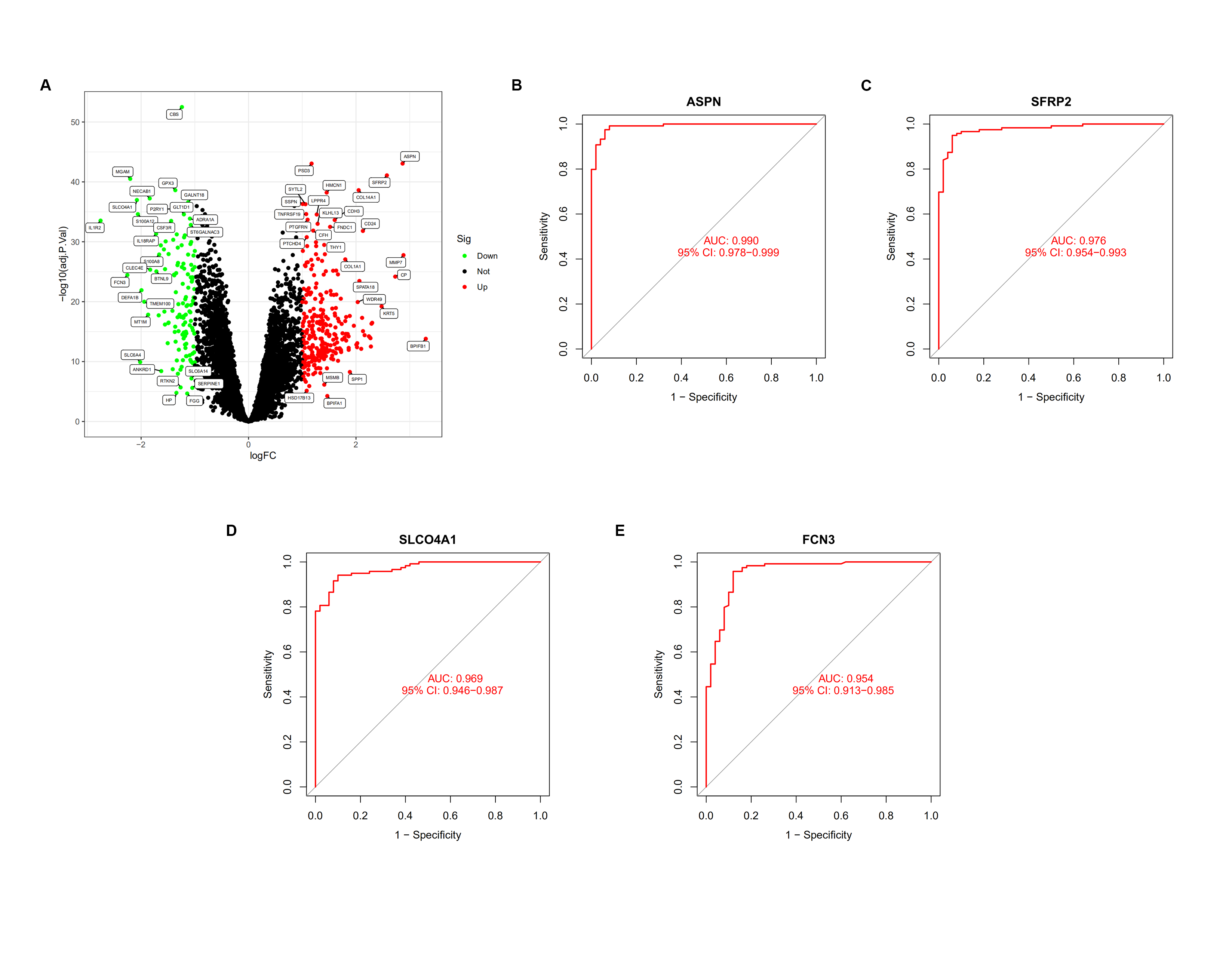

Supplement: Supplementary Figure 1 — (A) Volcano plot of DEGs between IPF and healthy control samples. (B-E) ROC curve for each candidate gene (ASPN, SFRP2, SLCO4A1 and FCN3). [file Image_1.tif]

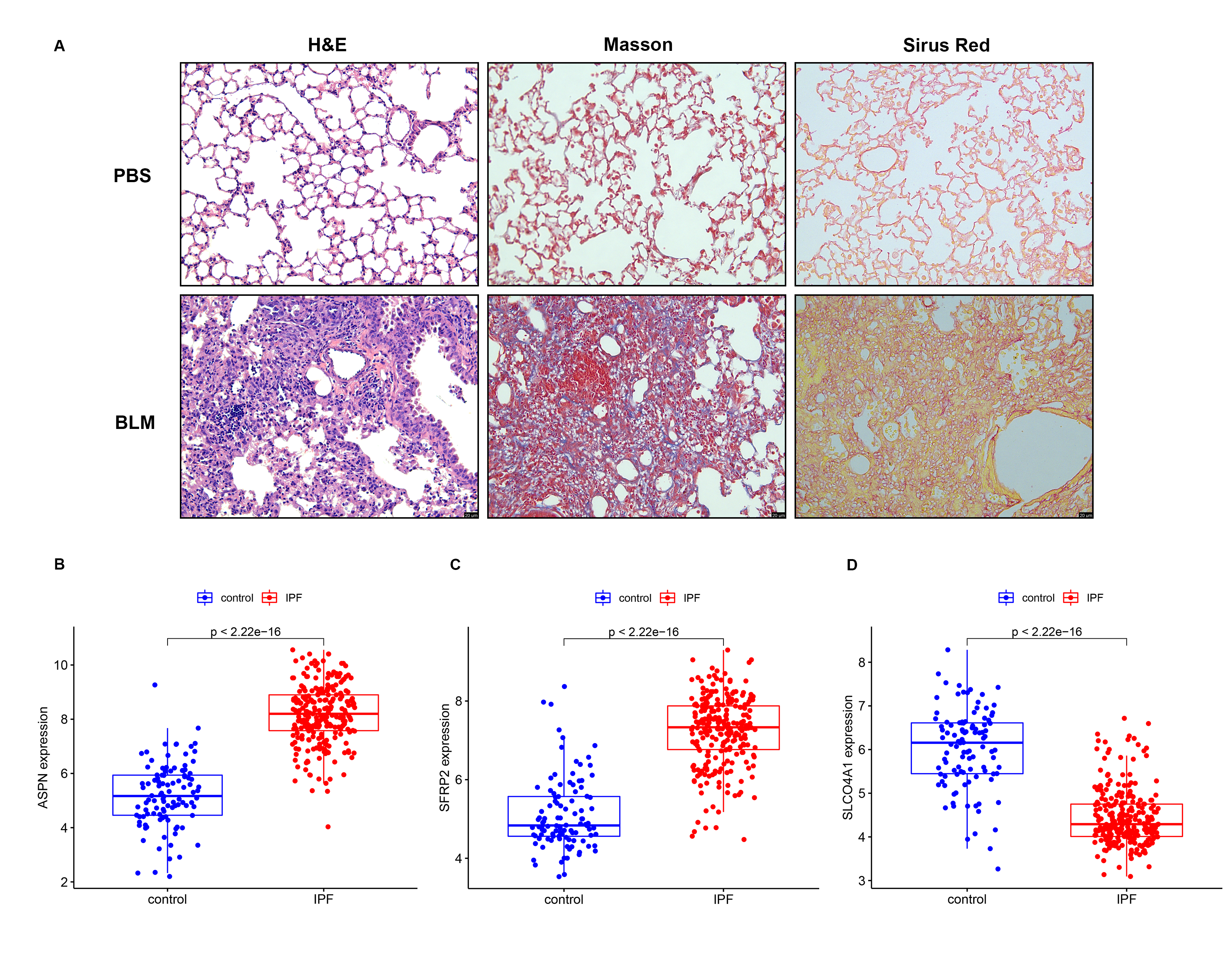

Supplement: Supplementary Figure 2 — (A) Representative hematoxylin and eosin (H&E), Masson’s trichrome and Sirius Red staining images obtained for the lungs of bleomycin-treated or PBS-treated mice. (B–D) Differential analysis of mRNA (ASPN, SFRP2 and SLCO4A1) expression levels using the meta-GEO cohort. [file Image_2.tif]

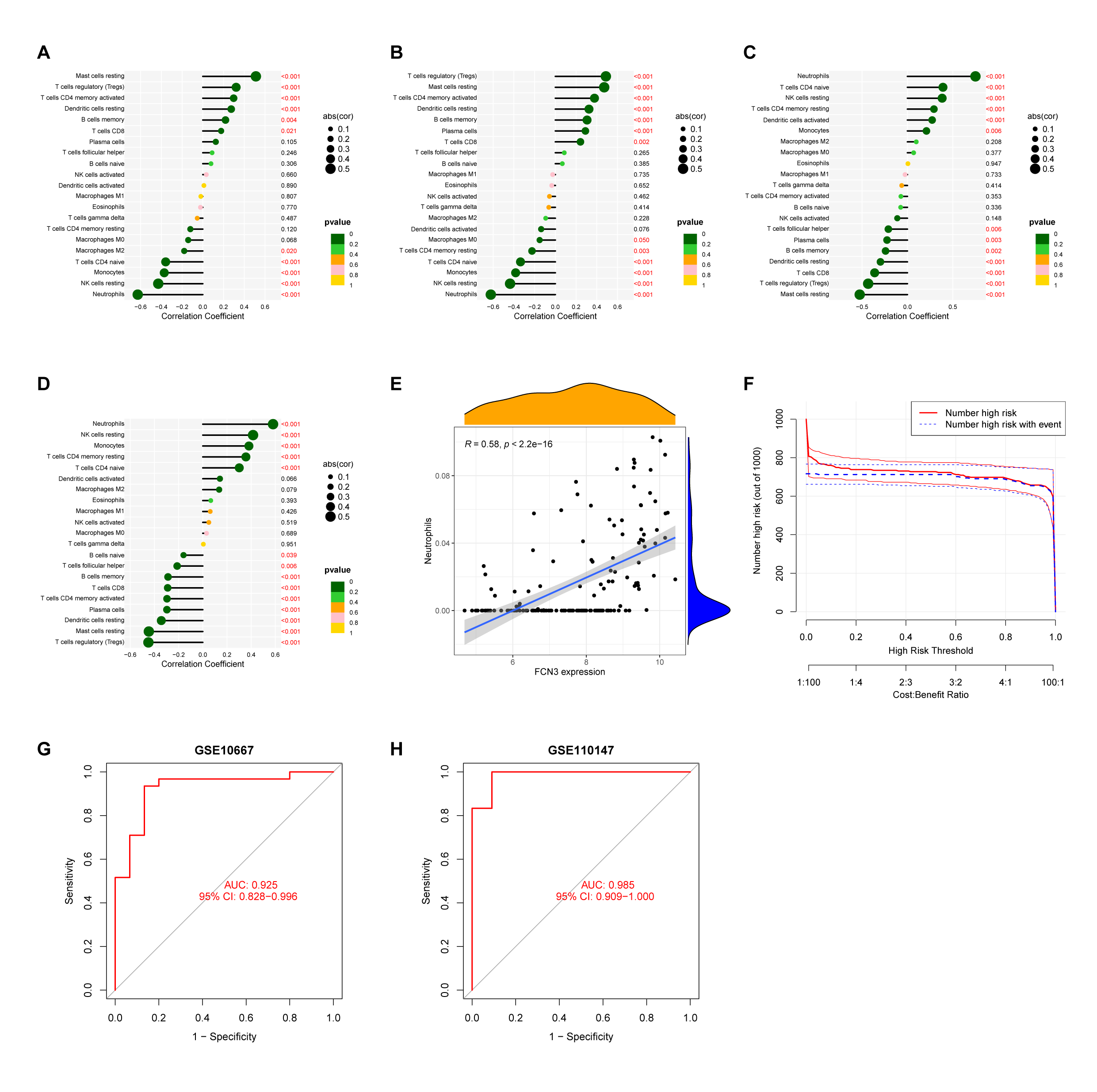

Supplement: Supplementary Figure 3 — (A-D) Correlation analysis between infiltrating immune cells and candidate hub genes (ASPN, SFRP2, SLCO4A1 and FCN3). (E) Correlation analysis between neutrophils and FCN3. (F) Clinical impact curve (CIC) of the diagnostic model. (G) ROC curve of an external validation cohort (GSE110147). (H) ROC curve of an external validation cohort (GSE10667). [file Image_3.tif]

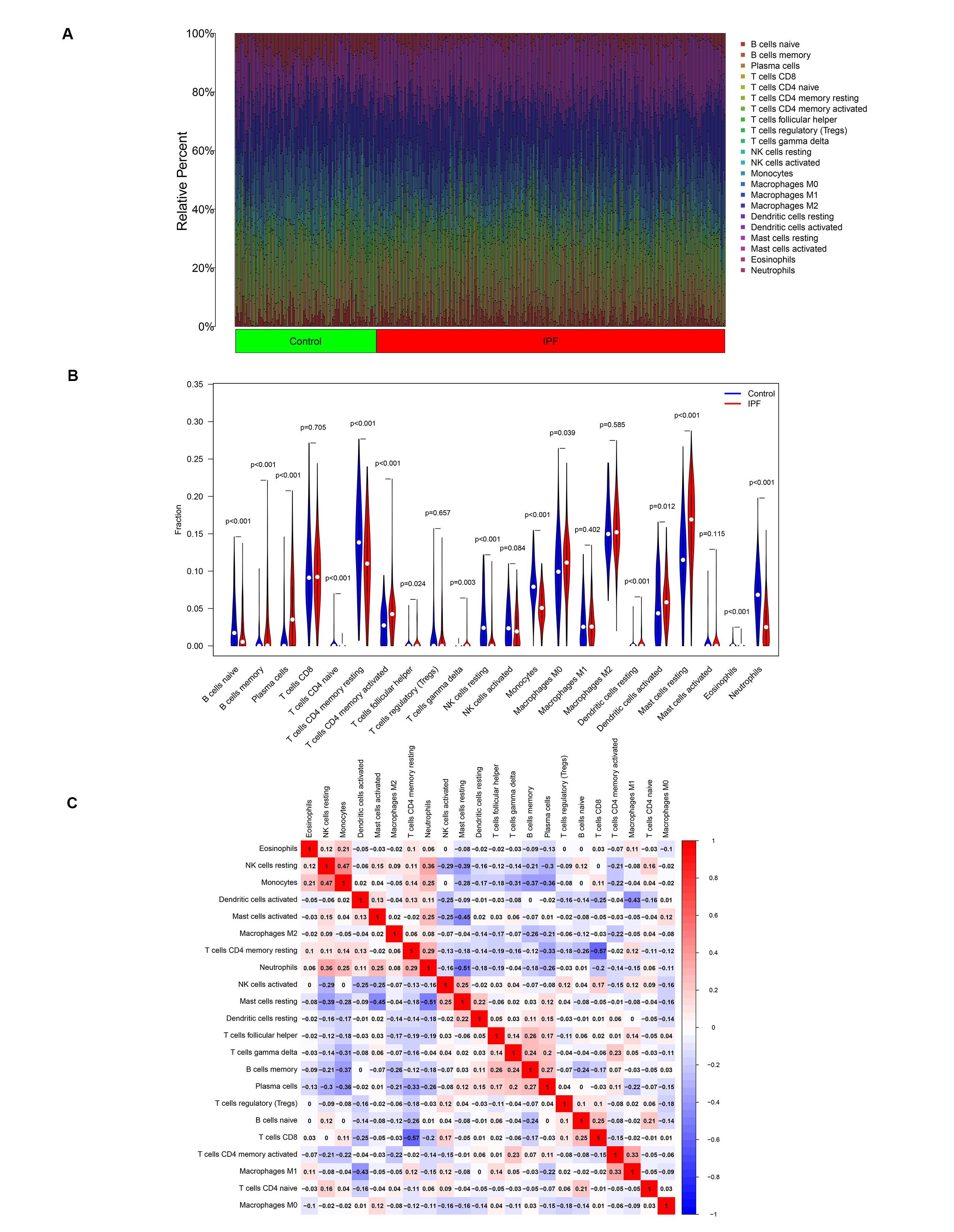

Supplement: Supplementary Figure 4 — Analysis of infiltrating immune cells in IPF using the meta-GEO cohort. (A) The proportions of different immune cells in IPF or healthy control lung samples. (B) Differential analysis of infiltrating immune cells between IPF and healthy control lung samples. (C) Correlation analysis of different infiltrating immune cells in the immune microenvironment of IPF. [file Image_4.tif]
