# Supplementary material for: Identification of diagnostic hub genes related to neutrophils and infiltrating immune cell alterations in idiopathic pulmonary fibrosis
Source: Front Immunol. 2023 Jun 2;14:1078055. doi: 10.3389/fimmu.2023.1078055 (PMC10272521; doi:10.3389/fimmu.2023.1078055)
Supplement: Supplementary file 5 [file Table_1.docx]

**Supplementary Tables**

**Supplementary Tables 1.** Primary and secondary antibodies.

| **Product** | **Catalog Number** | **Supplier** |
| --- | --- | --- |
| **Primary antibody:** |  |  |
| rabbit anti-Asporin | PA5-28124 | Invitrogen |
| rabbit anti-SFRP2 | DF4451 | Affinity Biosciences |
| rabbit anti-SLCO4A1 | XY12713 | XY-Bioscience |
| rabbit anti-GADPH | D16H11 | Cell Signaling Technology |
| rabbit anti-Ly6G | GB11229 | Servicebio |
| **Secondary antibody:** |  |  |
| anti-rabbit IgG HRP-linked Ab | 7074 | Cell Signaling Technology |
| goat anti-rabbit IgG Alexa 555 | A21428 | Invitrogen |

**Supplementary Tables 2.** Primer used for q-PCR.

| **Gene** | **Sequence (5′ to 3′)** | **Application** |
| --- | --- | --- |
| m*Aspn* | Forward: 5’-TCCTCTGACAAGGTTGGACT-3’ | qPCR |
|  | Reverse: 5’-AGAGAGTTGTCGTCATCATCGT-3’ |  |
| m*Sfrp2* | Forward: 5’-CGTGGGCTCTTCCTCTTCG-3’ | qPCR |
|  | Reverse: 5’-ATGTTCTGGTACTCGATGCCG-3’ |  |
| m*Slco4a1* | Forward: 5’-CGATCTGCACAGCTACCAGAG-3’ | qPCR |
|  | Reverse: 5’-GCTGACGAAGGTAAGGCATAG-3’ |  |
| m*18S* | Forward: 5’-GTGACGTTGACATCCGTAAAGA-3’ | qPCR |
|  | Reverse: 5’-GCCGGACTCATCGTACTCC-3’ |  |
